# Development and evaluation of serotype-specific recombinase polymerase amplification combined with lateral flow dipstick assays for the diagnosis of foot-and-mouth disease virus serotype A, O and Asia1

**DOI:** 10.1186/s12917-018-1644-4

**Published:** 2018-11-20

**Authors:** Hongmei Wang, Peili Hou, Guimin Zhao, Li Yu, Yu-wei Gao, Hongbin He

**Affiliations:** 1 Ruminant Diseases Research Center, Key Laboratory of Animal Resistant Biology of Shandong, College of Life Sciences, Shandong Normal University, Jinan, 250014 China; 2 Division of Livestock Infectious Diseases, State Key Laboratory of Veterinary Biotechnology, Harbin Veterinary Research Institute, Harbin, 150001 China; 3 Key Laboratory of Jilin Province for Zoonosis Prevention and Control, Military Veterinary Research Institute of Academy of Military Medical Sciences, Changchun, 130122 China

**Keywords:** FMDV, Serotype-specific, Recombinase polymerase amplification, Lateral flow dipstick

## Abstract

**Background:**

Foot-and-mouth disease (FMD) caused by foot-and-mouth disease virus (FMDV) is one of the most highly infectious diseases in livestock, and leads to huge economic losses. Early diagnosis and rapid differentiation of FMDV serotype is therefore integral to the prevention and control of FMD. In this study, a series of serotype-specific reverse transcription recombinase polymerase amplification assays combined with lateral flow dipstick (RPA-LFD) were establish to differentiate FMDV serotypes A, O or Asia 1, respectively.

**Results:**

The serotype-specific primers and probes of RPA-LFD were designed to target conserved regions of the FMDV VP1 gene sequence, and three primer and probe sets of serotype-specific RPA-LFD were selected for amplification of FMDV serotypes A, O or Asia 1, respectively. Following incubation at 38 °C for 20 min, the RPA amplification products could be visualized by LFD. Analytical sensitivity of the RPA assay was then determined with ten-fold serial dilutions of RNA of VP1 gene and the recombinant vector respectively containing VP1 gene from FMDV serotypes A, O or Asia1, the detection limits of these assays were 3 copies of plasmid DNA or 50 copies of viral RNA per reaction. Moreover, the specificity of the assay was assessed, and there was no cross reactions with other viruses leading to bovine vesicular lesions. Furthermore, 126 clinical samples were respectively detected with RPA-LFD and real-time PCR (rPCR), there was 98.41% concordance between the two assays, and two samples were positive by RPA-LFD but negative in rPCR, these were confirmed as FMDV-positive through viral isolation in BHK-21 cells. It showed that RPA-LFD assay was more sensitive than the rPCR method in this study.

**Conclusion:**

The development of serotype-specific RPA-LFD assay provides a rapid, sensitive, and specific method for differentiation of FMDV serotype A, O or Asia1, respectively. It is possible that the serotype-specific RPA-LFD assay may be used as a integral protocol for field detection of FMDV.

## Background

Foot-and-mouth disease (FMD) is an acute, highly contagious disease that affects cloven-hoofed animals, often resulting in huge economical losses in terms of trade and animal productivity. Recent outbreaks of FMD in Taiwan, Japan, South Korea and the United Kingdom have directly caused the culling of millions of animals, compensated heavily by the government [1, 2]. The responsible virus, FMD virus (FMDV), was a single-stranded positive-sense RNA virus belonging to the Aphthovirus genus in the family Picornaviridae. There were seven serotypes including O, A, C, Asia 1, and South African Territories (SAT) 1, 2 and 3, which together manifest a distinct geographical distribution [3]. FMDV serotype A, C and O are widely distributed across the world while Asia 1 and SAT 1–3 mainly occur in Asia and Africa, respectively. Several outbreaks of FMD serotype Asia 1, O and A have been recorded in mainland provinces of Southern China during 1999–2013 [4–6]. Early diagnosis of FMDV is therefore essential to providing valuable epidemiological information, and initiating the appropriate prevention and control strategies.

FMDV can be detected from blood, esophageal-pharyngeal fluid, nasal fluid, saliva, and other excretions of FMDV infected animals before clinical symptoms [7, 8] start to show. Currently, there are three typical assays for FMDV diagnosis including virus isolation, antigen enzyme-linked immunosorbent assay (Ag-ELISA) and real-time RT-PCR (rRT-PCR) used in FMDV reference laboratories [9]. However, these diagnostic tests require special equipment and professionally trained personnel. Another alternative propose is to use isothermal assays for diagnosis of FMDV. To date, there are four isothermal assays to detect FMDV: reverse transcription loop-mediated isothermal amplification (RT-LAMP) [10, 11], reverse transcription recombinase polymerase amplification (RT-RPA) [12], and nucleic acid sequence based amplification [13, 14]. RT-LAMP and RT-RPA have also been used to distinguish various serotypes in clinical samples [11, 12]. However, LAMP assay needs more primers than PRA, leading to longer amplicons and difficult designs in cases of highly variable viruses.

The RPA method is probably the one promising direction capable of rapid diagnosis of many different pathogens [12, 15–18]. The amplification relies on recombinase, single stranded binding protein, and strand displacing DNA polymerase at a constant temperature. The RPA products could be analyzed with gel electrophoresis, fluorescence monitoring based on probes, or simple visualization with a lateral flow dipstick (LFD) [19–22].

In the present study, a reverse transcription serotype-specific RPA-LFD assay was established, and evaluated as a field method for diagnosis and typing of FMDV serotypes A, Asia 1 or O, respectively.

## Results

### Design and optimization of serotype-specific RPA primers and probe

**Fig. 1:**
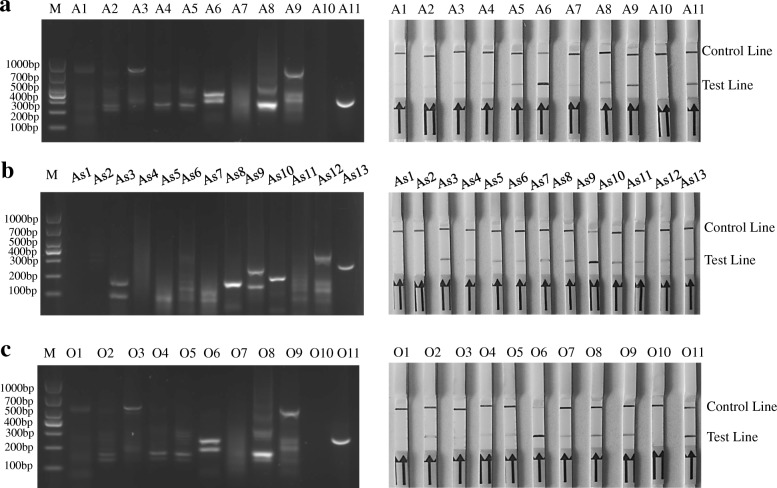
Screening of the primers/probes for the FMDV serotype-specific RPA-LFD assay. **a** Agarose gel electrophoresis and LFD detection of RT-RPA products amplified with different primer and probe sets of FMDV serotype A. Lane M was DNA Marker DL1000. A1 to A9 were different primer and probe sets. A6: the optimal primer and probe set, and the estimated size of the RPA amplified fragment were 346 bp and 334 bp. A10: negative control, (DNase-free water). A11: positive control (supplied by Twist Amp nfo kit). **b** Agarose gel electrophoresis and LFD detection of RT-RPA products amplified with different primers/probe sets of FMDV serotype Asia 1. As2 to As10 were different primer and probe sets. As9: the optimal primer/probe set, and the estimated size of the RPA amplified fragment were 286 bp and 182 bp. As1: negative control (DNase-free water). As10: positive control (supplied by Twist Amp nfo kit). **c** Agarose gel electrophoresis and LFD detection of RT-RPA products amplified with different primers/probe sets of FMDV serotype O. O1 to O9 were different primer and probe sets. O6: the optimal primer/probe set, and the estimated size of the RPA amplified fragment were 231 bp and 190 bp. O10: negative control (DNase-free water). O11: positive control (supplied by Twist Amp nfo kit)

The TwistAmp nfo reactions were performed to screen the candidate primer/probes for the RPA-LFD assay, the products were analyzed on 2% agarose gel. A6, As9, O6 sets of the primer/probes were respectively screened as serotype-specific RPA primers and probe for FMDV serotypes A, Asia-1, or O. They respectively produced 346 bp and 334 bp, 286 bp and 182 bp, 231 bp and 190 bp amplification products and their RPA-LFD test line appeared faster and darker than other sets within 5 min (Table 1 and Fig. 1).

**Table 1:** The sequences of primers and probes designed for screening in the study

Primer/probe set Name		Sequence(5′ → 3′)	The location on accession number	Product sizes (bp)
	F2	ATGGAGCACCTGAGGCAGCACTGGACAACA	3452–3481	221
A1	P1	[FAM]CACTGGACAACACGAGCAACCCCACTGCTTA[dSpacer] TATAAAGCACCGTTCACA[C3-spacer]	3470–3519	203
	R1	[Biotin]CGTTGAGAAGGGCACAGTCGTATTGAAACA	3643–3672	
	F2	ATGGAGCACCTGAGGCAGCACTGGACAACA	3452–3481	258
A2	P1	[FAM]CACTGGACAACACGAGCAACCCCACTGCTTA[dSpacer] TATAAAGCACCGTTCACA[C3-spacer]	3470–3519	250
	R2	[Biotin]GCACGAGGAGTTCTTGGATCTCCGTGGCTC	3680–3709	
	F2	ATGGAGCACCTGAGGCAGCACTGGACAACA	3452–3481	352
A3	P1	[FAM]CACTGGACAACACGAGCAACCCCACTGCTTA[dSpacer] TATAAAGCACCGTTCACA[C3-spacer]	3470–3519	229
	R3	[Biotin]GCAGGGGCAATAATTTTCTGCTTGTGTCTG	3774–3803	
	F1	CACCTGAGGCAGCACTGGACAACACGAGCAA	3458–3488	215
A4	P1	[FAM]CACTGGACAACACGAGCAACCCCACTGCTTA[dSpacer] TATAAAGCACCGTTCACA[C3-spacer]	3470–3519	203
	R1	[Biotin]GCCGTAGTTGAAGGAGGCAGGAAGCTGTGC	3643–3672	
	F1	CACCTGAGGCAGCACTGGACAACACGAGCAA	3458–3488	252
A5	P1	[FAM] CACTGGACAACACGAGCAACCCCACTGCTTA[dSpacer] TATAAAGCACCGTTCACA[C3-spacer]	3470–3519	240
	R2	[Biotin]GCACGAGGAGTTCTTGGATCTCCGTGGCTC	3680–3709	
A6 (Optimal set)	F1	CACCTGAGGCAGCACTGGACAACACGAGCAA	3458–3488	346
	P1	[FAM]CACTGGACAACACGAGCAACCCCACTGCTTA[dSpacer] TATAAAGCACCGTTCACA[C3-spacer]	3470–3519	334
	R3	[Biotin]GCAGGGGCAATAATTTTCTGCTTGTGTCTG	3774–3803	
	F3	TGAGGCAGCACTGGACAACACGAGCAACCC	3462–3461	211
A7	P1	[FAM]CACTGGACAACACGAGCAACCCCACTGCTTA[dSpacer] TATAAAGCACCGTTCACA[C3-spacer]	3470–3519	203
	R1	[Biotin]GCCGTAGTTGAAGGAGGCAGGAAGCTGTGC	3643–3672	
	F3	TGAGGCAGCACTGGACAACACGAGCAACCC	3462–3461	248
A8	P1	[FAM]CACTGGACAACACGAGCAACCCCACTGCTTA[dSpacer] TATAAAGCACCGTTCACA[C3-spacer]	3470–3519	240
	R2	[Biotin]GCACGAGGAGTTCTTGGATCTCCGTGGCTC	3680–3709	
	F3	TGAGGCAGCACTGGACAACACGAGCAACCC	3462–3461	342
A9	P1	[FAM]CACTGGACAACACGAGCAACCCCACTGCTTA[dSpacer] TATAAAGCACCGTTCACA[C3-spacer]	3470–3519	334
	R3	[Biotin]GCAGGGGCAATAATTTTCTGCTTGTGTCTG	3774–3803	
	F1	AAAAGCAACCCATTACCCGCCTGGCACTCC	3582–3611	285
As1	P1	[FAM]ACCCGCCTGGCACTCCCTTACACCGCTCCC[dSpacer]ACCGTGTGCTTGCAACAGT[C3-spacer]	3596–3645	271
	R2	[Biotin]GACTCTTCCCCGTAGGTTGTCTTCCCGTTG	3837–3866	
As2	F1	AAAAGCAACCCATTACCCGCCTGGCACTCC	3582–3611	145
	P1	[FAM]ACCCGCCTGGCACTCCCTTACACCGCTCCC[dSpacer]ACCGTGTGCTTGCAACAGT[C3-spacer]	3596–3645	131
	R3	[Biotin]GGGAGTGCCAGGCGGGTAATGGGTTGCTTT	3697–3726	
	F2	AACCCAACCGCCTACCAAAAGCAACCCATT	3566–3595	153
As3	P1	[FAM]ACCCGCCTGGCACTCCCTTACACCGCTCCC[dSpacer]ACCGTGTGCTTGCAACAGT[C3-spacer]	3596–3645	123
	R1	[Biotin]CGGTGTAAGGGAGTGCCAGGCGGGTAATGG	3689–3718	
	F2	AACCCAACCGCCTACCAAAAGCAACCCATT	3566–3595	301
As4	P1	[FAM]ACCCGCCTGGCACTCCCTTACACCGCTCCC[dSpacer]ACCGTGTGCTTGCAACAGT[C3-spacer]	3596–3645	271
	R2	[Biotin]GACTCTTCCCCGTAGGTTGTCTTCCCGTTG	3837–3866	
	F2	AACCCAACCGCCTACCAAAAGCAACCCATT	3566–3595	161
As5	P1	[FAM]ACCCGCCTGGCACTCCCTTACACCGCTCCC[dSpacer]ACCGTGTGCTTGCAACAGT[C3-spacer]	3596–3645	131
	R1	[Biotin]CGGTGTAAGGGAGTGCCAGGCGGGTAATGG	3689–3718	
	F3	CGAATCAGCAGACCCAGTTACCACCACAGT	3274–3303	445
As6	P2	[FAM]TGAAACTCACACAGCTCAAGAACACCCAAACT[dSpacer] TTGATCTTATGCAAATC[C3-spacer]	3378–3427	341
	R1	[Biotin]CGGTGTAAGGGAGTGCCAGGCGGGTAATGG	3689–3718	
	F3	CGAATCAGCAGACCCAGTTACCACCACAGT	3274–3303	593
As7	P2	[FAM]TGAAACTCACACAGCTCAAGAACACCCAAACT[dSpacer] TTGATCTTATGCAAATC[C3-spacer]	3378–3427	489
	R2	[Biotin]GACTCTTCCCCGTAGGTTGTCTTCCCGTTG	3837–3866	
	F3	CGAATCAGCAGACCCAGTTACCACCACAGT	3274–3303	453
As8	P2	[FAM]TGAAACTCACACAGCTCAAGAACACCCAAACT[dSpacer] TTGATCTTATGCAAATC[C3-spacer]	3378–3427	349
	R3	[Biotin]GGGAGTGCCAGGCGGGTAATGGGTTGCTTT	3697–3726	
As9 (Optimal set)	F3	CGAATCAGCAGACCCAGTTACCACCACAGT	3274–3303	286
	P2	[FAM]TGAAACTCACACAGCTCAAGAACACCCAAACT[dSpacer] TTGATCTTATGCAAATC[C3-spacer]	3378–3427	182
	R4	[Biotin]GAGAAGTAGTACGTCGCAGACCGAAGTAGCG	3530–3559	
	F3	CGAATCAGCAGACCCAGTTACCACCACAGT	3274–3303	445
As10	P1	[FAM]ACCCGCCTGGCACTCCCTTACACCGCTCCC[dSpacer]ACCGTGTGCTTGCAACAGT[C3-spacer]	3378–3427	341
	R1	[Biotin]CGGTGTAAGGGAGTGCCAGGCGGGTAATGG	3689–3718	
	F3	CGAATCAGCAGACCCAGTTACCACCACAGT	3274–3303	593
As11	P1	[FAM]ACCCGCCTGGCACTCCCTTACACCGCTCCC[dSpacer]ACCGTGTGCTTGCAACAGT[C3-spacer]	3596–3645	271
	R2	[Biotin]GACTCTTCCCCGTAGGTTGTCTTCCCGTTG	3837–3866	
	F1	CAACACCACCAACCCAACGGCGTACCATAA	3570–3599	161
O1	P1	[FAM]CGTACCATAAGGCGCCGCTTACCCGGCTTA[dSpacer] ATTGCCCTACACGGCACCA[C3-spacer]	3590–3639	141
	R1	[Biotin]GAGCCAGCACTTGGAGATCGCCTCTCACGT	3701–3730	
	F1	CAACACCACCAACCCAACGGCGTACCATAA	3570–3599	343
O2	P1	[FAM]CGTACCATAAGGCGCCGCTTACCCGGCTTA[dSpacer] ATTGCCCTACACGGCACCA[C3-spacer]	3590–3639	323
	R2	[Biotin]CAAGGACTGCTTTACAGGTGCCACTATTTT	3883–3912	
	F1	CAACACCACCAACCCAACGGCGTACCATAA	3570–3599	210
O3	P1	[FAM]CGTACCATAAGGCGCCGCTTACCCGGCTTA[dSpacer] ATTGCCCTACACGGCACCA[C3-spacer]	3590–3639	190
	R3	[Biotin]TTGATGGCACCGTAGTTGAAAGAAGTAGGC	3751–3779	
	F2	GGAGCACCTGAAGCAGCCTTGGACAACACC	3549–3578	182
O4	P1	[FAM]CGTACCATAAGGCGCCGCTTACCCGGCTTA[dSpacer] ATTGCCCTACACGGCACCA[C3-spacer]	3590–3639	141
	R1	[Biotin]GAGCCAGCACTTGGAGATCGCCTCTCACGT	3701–3730	
	F2	GGAGCACCTGAAGCAGCCTTGGACAACACC	3549–3578	364
O5	P1	[FAM]CGTACCATAAGGCGCCGCTTACCCGGCTTA[dSpacer] ATTGCCCTACACGGCACCA[C3-spacer]	3590–3639	323
	R2	[Biotin]CAAGGACTGCTTTACAGGTGCCACTATTTT	3883–3912	
O6 (Optimal set)	F2	GGAGCACCTGAAGCAGCCTTGGACAACACC	3549–3578	231
	P1	[FAM]CGTACCATAAGGCGCCGCTTACCCGGCTTA[dSpacer] ATTGCCCTACACGGCACCA[C3-spacer]	3590–3639	190
	R3	[Biotin]TTGATGGCACCGTAGTTGAAAGAAGTAGGC	3751–3779	
	F3	GGGGACCTTACCTGGGTGCCAAATGGAGCA	3524–3553	217
O7	P2	[FAM]CAAATGGAGCACCTGAAGCAGCCTTGGACAA[dSpacer]ACCACCAACCCAACGGCGTAC[C3-spacer]	3542–3596	189
	R1	[Biotin]GAGCCAGCACTTGGAGATCGCCTCTCACGT	3701–3730	
	F3	GGGGACCTTACCTGGGTGCCAAATGGAGCA	3524–3553	389
O8	P2	[FAM]CAAATGGAGCACCTGAAGCAGCCTTGGACAA[dSpacer]ACCACCAACCCAACGGCGTAC[C3-spacer]	3542–3596	371
	R2	[Biotin]CAAGGACTGCTTTACAGGTGCCACTATTTT	3883–3912	
	F3	GGGGACCTTACCTGGGTGCCAAATGGAGCA	3524–3553	256
O9	P2	[FAM]CAAATGGAGCACCTGAAGCAGCCTTGGACAA[dSpacer]ACCACCAACCCAACGGCGTAC[C3-spacer]	3542–3596	238
	R3	[Biotin]TTGATGGCACCGTAGTTGAAAGAAGTAGGC	3751–3779	

Note: *F*:forward primer, *R* reverse primer, *P* probe, *FAM* Carboxyfluorescein, *dSpacer* A tetrahydrofuran residue, *C3-spacer* 3’-block

**Fig. 2:**
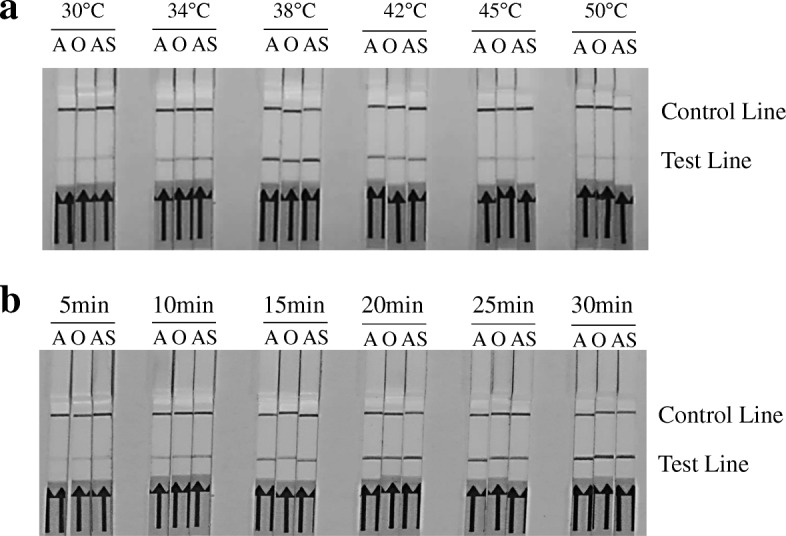
Optimization of reaction temperature and time for FMDV serotype-specific RPA-LFD assays. **a** The RPA-LFD performs effectively in a wide range of constant reaction temperatures. **b** The amplified products can be visible on the LFD at 5 min or longer

Besides that, different reaction conditions were optimized for the serotype-specific RPA assay. Results showed that a reaction temperature at 38 °C (Fig. 2a) and a incubation time of 20 min or longer (Fig. 2b) can best promote the amplification efficiency. Thus, the amplification reaction for the serotype-specific RPA assay should be carried out at 38 °C for 20 min.

### Sensitivity and specificity of the FMDV serotype-specific RPA reaction

**Fig. 3:**
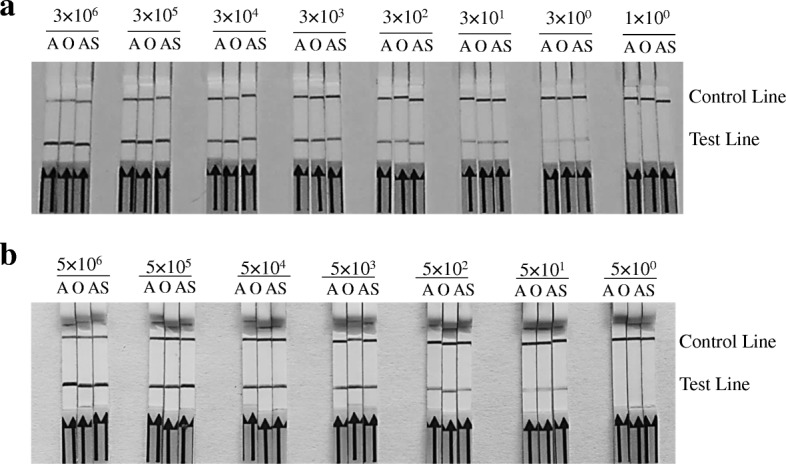
The sensitivity of FMDV serotype-specific RPA-LFD assays. **a** Sensitivity of the standard plasmids. Molecular sensitivity of RPA-LFD was determined using 10-fold serially diluted 3 × 10^6^ to 3 × 10^0^ copies and 10^0^ copy of FMDV DNA standard plasmids per reaction as template. The minimum limits for virus detection of RPA-LFD were 3 × 10^0^ copies. **b** Sensitivity of the RNA standard. The cDNA of reverse transcription using 10-fold serially diluted 5 × 10^6^ to 5 × 10^0^ RNA molecular was used in molecular sensitivity of RPA-LFD. The minimum limits detection of RPA-LFD were 5 × 10^0^ RNA. A: primers/probe set of FMDV serotype A. AS: primers/probe sets of FMDV serotype Asia 1. O: primers/probe sets of FMDV serotype O. Samples were tested in triplicate with one reaction and independently repeated 3 times

To determine the sensitivity of RPA assay, positive vector and RNA standard of three FMDV serotypes were diluted from 3 × 10^6^ to 3 × 10^0^ copies/μL and 5 × 10^7^ to 5 × 10^1^ molecules/μL, respectively. RNA was reversely transcribed to cDNA. All reverse transcriptional cDNA and positive vectors of each dilution were respectively used as templates in the RPA reactions. All RPA reaction products were than respectively tested on LFD, and the limit of detection with the RPA-LFD was 3 × 10^0^ dilution for positive vector (Fig. 3a) and 5 × 10^1^ dilution for RNA standard (Fig. 3b).

**Fig. 4:**
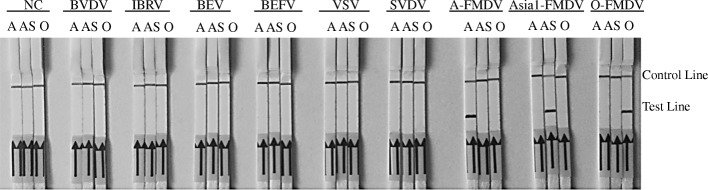
The specificity of the FMDV RPA-LFD assays. Other bovine viral pathogens with similar clinic and etiologies were used to assess the specificity of the assays. There was no cross-reaction with BVDV, IBRV, BEV, BEFV, BVSV and SVDV. NC: negative control. A: primers/probe set of FMDV serotype A. AS: primers/probe set of FMDV serotype Asia 1. O: primers/probe set of FMDV serotype O. Samples were tested in triplicate with one reaction and three separate assays

The other viral pathogens with similar clinical signs, including BEV, BVDV, BEFV, VSV, IBRV, and SVDV, were used to assess the specificity of the assay. IBRV DNA and other viral cDNA were respectively detected with the RPA-LFD. There was no cross reaction with the other bovine viral pathogens with similar clinical signs (Fig. 4). The cDNA of other FMDV epidemic virus strains in China were used for detection of serotype-specific RPA-LFD. There was no cross-reactivity with different serotype strains of FMDV, so the specific primer and probe sets could differentiate the corresponding serotype virus.

### Performance of FMDV serotype-specific RPA-LFD assay on clinical samples

To evaluate the diagnostic sensitivity of the FMDV RPA-LFD assay, cDNA obtained from each specimen of 126 clinical samples were detected with RPA-LFD and rPCR, respectively. RPA-LFD identified 41 negative and 85 positive samples (25 vesicular material, 14 saliva, 10 aerosol, 14 oesophageal-pharyngeal fluid, 9 blood and 13 nasal swabs) (Table 2). Of the 85 positive samples, 32 were serotyped as serotype A, 17 as serotype Asia 1, and 36 as serotype O. The concordance between FMDV RPA-LFD and rPCR was 98.41% (124/126).

**Table 2:** Comparative performance of serotype-specific RT-LFD-RPA and rRT-PCR assays for detection of suspected clinical specimens and serotyping of FMDV

				LFD-RPA					Real-time	(qPCR)	
Samples name	A	Asia 1	O	FMDV positive	FMDV Negative	A	Asia 1	O	FMDV positive		FMDV Negative
vesicular material	7	3	15	25	5	7	3	15	25		5
nasal swab	6	2	5	13	7	6	2	5	13		7
saliva	5	3	6	14	15	5	3	6	14		15
oesophageal pharyngeal fluid	6	4	4	14	3	6	4	4	14		3
blood	4	2	3	9	7	4	2	3	9		7
aerosol	4	3	3	10	4	3	3	2	8		6
Total	32	17	36	85	41	31	17	35	83		43

It is worth noting that 4 aerosol specimens of FMDV serotype A were designated as positive by the RPA-LFD assay, but only 3 of them were FMDV serotype A positive detected by the rPCR, and 3 aerosol specimens of FMDV serotype O were differentiated with the RPA-LFD assay, whereas 2 of them were FMDV serotype O positive using the rPCR. BHK-21 cells were used to isolate viruses and identify the two inconsistent aerosol specimens, and CPE of cells inoculated with two inconsistent samples appeared untill the third passage. CPE-positive cell and control cell culture were respectively harvested and detected for FMDV using rPCR and RPA-LFD. As expected, CPE-positive cells were respectively identified for FMDV serotype O and A, and the control cells were FMDV negative, it showed that the sensitivity of RPA-LFD assay was higher than the rPCR in this study.

## Discussion

There are a series of methods used as detection of FMDV, such as Ag-ELISA, virus separation, and rRT-PCR. However, the above methods have been either too time-consuming or require high-precision instruments to meet practical needs [19, 20]. RPA isothermal amplification techniques can amplify nucleic acids and detect the products without a requirement of special instrument or complex operations [21–24]. It is worth mentioning that human body heat can indeed incubate RPA reactions under certain limit resources [25]. Moreover, it is not required to store the lyophilized RPA reagents with a cooling chain because they can actually be stably stored at room temperature for a longer time [26].

Although it has been proved that the pan-specific real-time RT-RPA (rRT-RPA) and RPA-LFD technique can provide rapid and accurate diagnosis of FMDV [12, 18], they are difficult to distinguish the serotypes of FMDV for the rRT-RPA assay. The VP1, a surface exposed-capsid protein, take a pivotal role in the antigenicity as a major viral antigen, and plays an important role in pathogenicity of FMDV as its binding to viral receptors of host cells. Because of heterogeneity, the nucleotide sequence encoding VP1 is widely used to determine genetic relationships between different strains and to trace the provenance and transmission route of epidemic FMDV strains [27–30]. In this study, primer and probe sets specific for serotypes O, A, or Asia-1 FMDV were designed based on the alignment of the nucleotide sequences of viral VP1 gene of the above serotypes strains circulating in Asia, respectively. The primer and probe sets A6, As9, and O6 screened for RPA in this study could perform effective and accurate detection of different FMDV serotype of A/China/5/99, Asia1/AF/72, and O/HNK/CHA/05 (see Fig. 1) as intended. Furthermore, the serotype-specific RPA-LFD assay successfully detected the epidemic strains of FMDV in China (Table 2), and provided a more robust assessment method regarding the serotype specificity.

FMDV is mainly transmitted by aerosol. Viral RNA was detected in aerosol samples from FMDV suspected farm at 1–3 days before infected cattle appeared clinical signs [31]. Based on the aerogenous characteristics of the FMDV, it’s proven to be a valuable technique that aerosol samples were used to detect viral RNA in infected farms [31, 32]. In our study, viruses in aerosol were also detected and their serotypes were respectively differentiated by the FMDV RPA-LFD assay. Therefore, it may be the potential integral monitoring strategies for prevention, control and eradication of FMD using this technique.

It is a crucial step for clinical detection with any molecular diagnostic assay that the nucleic acids are extracted from tissue and cell samples, the Punch-it™ kit can easily isolate nucleic acid from different samples via paper chromatography, and becomes one of the ideal tools for the extraction of DNA/RNA. Recent study showed that the DNA isolated with the Punch-it™ kit could be used in molecular assays [33]. In our study, nucleic acids of different samples, including vesicular material, blood, oesophageal-pharyngeal fluid, saliva, aerosol, and nasal swabs, were successfully extracted using the Punch-it™ kit. The method only takes 10 min to extract the nucleic acid without centrifugation, so it is relatively simple and rapid, and it is even more important that the extracted nucleic acids can directly serve as templates in RPA-LFD assays. In the present study, the extracted total RNA needed to be reverse transcribed into cDNA in the two-step RPA-LFD assay, whereas reverse transcription and RPA were performed in one reaction using the TwistAmp™ exo kit (TwistDx Limited, UK) and with the addition of reverse transcriptase in previously studied RT-RPA assays [12]. We would like to further simplify the test to make it more suitable for field use in future. Moreover, sophisticated instrumentations were required in RT-RPA [12, 15, 16], whereas RPA-LFD assay in this study only needs a thermos metal bath for incubation at 38 °C and amplified products can be direct visible on the LFD without requirements of instruments. Therefore, it may be an effective way to detect clinical samples in the field.

## Conclusions

In the present study, the serotype-specific FMDV RPA-LFD assay was successfully developed, and will be helpful for detection of FMDV infection during FMD outbreaks. Because RPA-LFD assay is a simple, specific, rapid, and serotype-specific method, it is possible to be a general differentiated protocol for diagnostics of FMDV, especially for detection of clinical samples in the field.

## Methods

### Virus and clinical specimens

In this study, cDNA of three serotypes of FMDV reference strains including type O strain China/5/99, type A strain AF/72, and type Asia 1 strain HNK/CHA/05, which were provided by Harbin Veterinary Research Institute, Chinese Academy of Agricultural Sciences, were positive controls of various serotypes used to optimize primer and probe sets of RPA. The cDNA of other epidemic FMDV strains in China including type A (HuBWH/2009, Mya98, GSLX/2010, GDMM/2013), type Asia1 (ZB/58, HeB/05, YS/05, HN/06, BR/Myanmar/06, WHN/06), and type O (LY/2000, CC/03, GZ/2010, BY/2010, HKN/2011, GD/2013, GD/2015), were used to provide a more robust assessment of serotype-specific RPA. Other viral pathogens causing similar clinical vesicular signs, including bovine ephemeral fever virus (BEFV), vesicular stomatitis virus (VSV), bovine viral diarrhea virus (BVDV), bovine enterovirus (BEV), infectious bovine rhinotracheitis virus (IBRV),and swine vesicular disease virus (SVDV), were stored by the Ruminant Diseases Research Center, Shandong Normal University, and used for cross-reactivity testing. To compare the detection sensitivity between RPA-LFD reactions and rPCR, 126 clinical specimens (30 vesicular materials, 29 salivas, 14 aerosols, 16 bloods, 17 oesophageal-pharyngeal fluids, and 20 nasal swabs) were collected from suspected cases of FMD in the Chinese endemic region from 2013 to 2017.

### Isolation of viral RNA/DNA and synthesis of cDNA

Viral RNA and DNA were isolated using the MiniBEST Viral RNA/DNA Extraction Kit (TaKaRa, Dalian, China) following respective instructions. The amounts of viral RNA was measured using a Biophotometer plus (Eppendorf, USA). The extracted RNA was template used to synthesized cDNA with reverse transcription using random primers in a total volume of 10 μL according to the instructions of the PrimeScript™ RT Master Mix (Takara, Dalian, China). All viral DNA and cDNA were stored at − 70 °C for further employment.

### Generation of DNA/RNA molecular standard

The viral VP1 gene recombinant vectors respectively containing RPA amplified region of FMDV serotype O, A, or Asia 1 (named pET32a-A-FMDV-VP1, pET32a-AS1-FMDV-VP1 and pET32a-O-FMDV-VP1) were constructed and used for the analytical sensitivity. The Positive plasmids were measured using a Biophotometer plus (Eppendorf, USA), respectively. The quantity of copies was calculated by the formula: DNA copy number (copies/μL) = (M × 6.02 × 10^23^ × 10^− 9^)/(n × 660), M: molecular weight, n:plasmid concentration (g/μL) measured at 260 nm.

RNA molecular standards were prepared as described in previous study [17] with certain modifications. The linearized recombinant vectors with SgrA I (New England Biolabs, USA) were purified with the MiniBEST DNA Fragment Purification Kit (Takara, Dalian, China), and then used as template for RNA transcription with the RiboMAX Large Scale RNA Production System-T7 (Promega, USA). Furthermore, the RNA was measured using the Quant-iTTM RiboGreen RNA Assay Kit (Thermo Fisher Scientific, Germany) according to the manufacturer’s instructions. The quantity of copies was calculated by the equation: Amount (copies/μL) = [RNA concentration (g/μL)/(transcript length in nucleotides× 340)] × 6.02 × 10^23^.

### Design of serotype-specific RPA primers and probe

A multiple sequence alignment of FMDV strains of serotype A, Asia 1, or O was respectively performed to find highly conserved region of the FMDV VP1 gene. The following reference sequences of three serotypes found in GenBank database were respectively used: KT968663(A/HY/CHA/2013), FJ755082 (A/PAK/1/2006), KY322679 (A/TAI/4/2014), FJ755052 (A/IRN/51/2005), KY404935 (A/A01NL), EF149010 (Asia 1/HNK/CHA/05), EF614458 (Asia1/MOG/05), AY687334 (Asia1/IND 491/97), GU931682 (Asia1/YS/CHA/05), AY687333 (Asia1/IND 321/01), HQ009509 (O/China/5/99), LC149720 (O/JPN/2010–362/3), JN998086 (O/GZ/CHA/2010), AF095876(O/Taipei-150). RT-RPA primers and probes specific for serotypes O, A or Asia-1 of FMDV were designed against the consensus sequence for this region and were synthesized by Sangon Biotech, respectively. RPA primers/probes were synthesized and labeled as described in previous study [18]. Oligonucleotide sequences of RPA primers and probes of specific serotype A, Asia-1, or O of FMDV are listed in Table 1 (accession numbers KT968663, EF149010 and HQ009509, respectively).

### FMDV serotype-specific RPA assays

Serotype-specific primers and probes were screened as described in previous study [25] with some modifications. In brief, RPA was performed using a TwistAmp™ nfo kit (TwistDx Limited, UK). The freeze-dried enzyme pellet was dissolved with 47.5 μLof solution containing 29.5 μL rehydration buffers, 2.1 μL forward and reverse primers (10 μM), 0.6 μL probe (10 μM), 11.2 μL of sterile water, 2 μL of cDNA of FMDV reference strains, and then 2.5 μL magnesium acetate (280 mM) was added. Assays were completed in a thermos metal bath at 38 °C for 20 min. The amplified products were then put through a 2% (*w*/*v*) agarose gel electrophoresis to screen the optimal and serotype-specific primer and probe sets. The optimal reaction conditions were determined by testing various reaction temperatures and incubation times.

LFD double label with anti-FAM gold conjugates and anti-Biotin antibodies (Milenia Biotec GmbH, Germany) were used to visualize the RPA amplified products. 1 μL of RPA products were diluted with 99 μL Dipstick Assay Buffer (Milenia Biotec GmbH, Germany), and then tested by LFD. FMDV serotype-specific positives are indicated by the visualization of both a test line and control line simultaneously perceptible on the LFDs after 5 min, while the negative reactions only generate a control line. The cDNA of other FMDV epidemic virus strains in China were used for evaluation of serotype-specific RPA-LFD.

### Sensitivity and specificity of the RPA-LFD assay

To determine the DNA analytical sensitivity of the RPA-LFD assay, the recombinant plasmids pET32a-A-FMDV-VP1, pET32a-As1-FMDV-VP1, and pET32a-O-FMDV-VP1 were respectively the standard DNA template of FMDV serotype A, Asia 1 and O. The RPA-LFD assays were performed with ten-fold serial dilutions of the recombinant vector ranging from 3 × 10^6^ to 3 × 10^0^ copies per microliter for respective serotypes. To detect the RNA analytical sensitivity, RNA standards of three FMDV serotypes were diluted from 5 × 10^7^ to 5 × 10^1^ molecules/μL. 10 μL RNA was used as template to synthesize cDNA in 20 μL reverse transcription reaction system using PrimeScript™ RT Master Mix (Takara, Dalian, China) in accordance with the manufacturer’s instructions. 2 μL cDNA of each dilution was used as a template in the RPA reactions. DNA plasmid/RNA samples were detected with three separate assays, respectively.

The specificity of the method was assessed using other viral pathogens with similar clinical symptoms, including BEFV, VSV, BVDV, BEV, IBRV, and SVDV. The IBRV DNA was extracted and viral cDNA were reverse transcribed from isolated other viral RNA. Positive controls and negative controls for RPA were constructed using recombinant vectors and RNase free water.

### Diagnosis of clinical specimens with FMDV serotype-specific RPA-LFD assays

To compare the diagnostic sensitivity between RPA-LFD and rPCR, 126 clinical specimens were collected from bovine farms suspected with infection of FMDV in China from 2013 to 2017 (details listed in Table 2). RNAs were isolated from the clinical samples using a Punch-it™ Kit (Nanohelix, Daejeon, South Korea) following manufacturers’ instructions. A 1 mm punched disk, containing the nucleic acids, was added with 10 μL of reverse transcript reaction system using PrimeScript™ RT Master Mix (Takara, Dalian, China), which contained 2 μL 5 × PrimeScript RT Master Mix and 8 μL RNase Free dH_2_O. Reverse transcription for each sample was completed in two tubes using a thermos metal bath at 37 °C for 15 min. 2 μL of cDNA was then used in both RPA-LFD and rPCR reactions. For the rPCR, serotype-specific primers and probes for serotypes O, A or Asia-1 FMDV were employed as previously described [23]. Reactions were performed with Premix Ex Taq™ Kit (Takara, Dalian, China) for respective serotypes.

Samples were positive in RPA-LFD, but negative in rPCR, were further tested for presence of FMDV. The virus was isolated using BHK-21 cells (provided by China Center for Type Culture Collection) as described in the previous study [18]. The cytopathic effect (CPE) was examined at 24 h, 48 h, and 72 h, respectively. If there was no CPE after 72 h, Cell cultures were passaged. CPE-positive, CPE-negative and control cell cultures were respectively harvested and examined again for FMDV using RPA-LFD and rPCR.

## Abbreviations

BEFV: Bovine ephemeral fever virus
BEV: Bovine enterovirus
BVDV: Bovine viral diarrhea virus
ELISA: Immune-histopathology and enzyme-linked immunosorbent assay
FMDV: Foot-and-mouth disease virus
IBRV: Infectious bovine rhinotracheitis virus
LAMP: Loop-mediated isothermal amplification
LFD: Lateral flow dipstick
PCR: Polymerase chain reaction
RPA: Recombinase polymerase amplification
rPCR: Real time PCR
RT: Reverse transcription
SAT: South African Territories
SVDV: Swine Vesicular Disease Virus
VSV: Vesicular stomatitis virus
